# Nurses’ Perceptions of the Quality of Perinatal Care Provided to Lesbian Women

**DOI:** 10.3389/fpsyg.2022.742487

**Published:** 2022-02-22

**Authors:** Sharona Tzur-Peled, Talma Kushnir, Orly Sarid

**Affiliations:** ^1^Dina Academic School of Nursing, Rabin Medical Center, Petah Tikva, Israel; ^2^Adelson School of Medicine, Department of Behavioral Sciences, Ariel University, Ariel, Israel; ^3^Department of Public Health, Ben-Gurion University of the Negev, Beersheba, Israel; ^4^The Spitzer Department of Social Work, Ben-Gurion University of the Negev, Beersheba, Israel

**Keywords:** attitudes, behavioral intentions, lesbians, perceptions of care, TRA

## Abstract

**Aim:**

Based on the Theory of Reasoned Action (TRA), we examined whether attitudes of nurses from different ethnic groups, subjective norms, behavioral intentions, assessments of relationships and communication were associated with their perceptions of the quality of perinatal care provided to lesbian women.

**Background:**

Nurses administer healthcare, provide pertinent information and consultation to lesbians from pregnancy planning through birth.

**Introduction:**

During the past few decades, worldwide, there has been a rise in lesbian-parenting. Despite the changes in Israeli society’s public and legal reality, intolerance and discrimination to the homosexual population is still prevalent in Israel’s healthcare system.

**Methods:**

A cross-sectional study conducted between 12/2015-4/2016. Of the 270 nurses approached, 184 completed a self-report anonymous questionnaire (a response rate of 74%).

**Findings:**

This is an important and timely study reflecting nurses’ perceptions of the quality of perinatal care provided to lesbians. The study findings reflect that attitudes, subjective norms, behavioral intentions, assessments of relationships and communication of nurses from different ethnic groups are associated with their perceptions of the quality of perinatal care provided to the lesbians. The hierarchical regression analysis demonstrated that attitudes, subjective norms, behavioral intentions, assessments of relationships and communication of nurses contributed 56% to the variance of nurses’ perception of their own quality of perinatal care.

**Discussion:**

TRA conceptualization predicted the quality of care of nurses from different ethnic groups treating lesbians in a perinatal setting.

**Conclusion:**

TRA provides a useful framework for understanding and predicting the motivational effect of health care personnel with the lesbian population, being at risk for stigmatization and receiving less quality perinatal care.

**Implications for nursing and heath policy:**

Our findings revealed the importance of formulating a recognized policy in the field of LGBT medicine at the national level. Further training of nurses as to the lesbians’ unique health needs, might improve the nurses’ relationships and communication as well as the quality of perinatal nursing care.

## Introduction

During the past few decades, same-sex parenthood in many countries has increased. In Israel, there are more than 2,500 lesbians raising children (Rosenblum and Peleg, 2007). Studies conducted in Israel and worldwide reveal that most lesbians choose to conceive through sperm donation (Bucholz, 2000; Ben-Ari and Livni, 2006). Consequently, these women undergo fertility treatments and receive perinatal health care at women’s health centers in the community. Nurses usually accompany the lesbian patients from the pregnancy planning stage through birth and the postpartum period and are available for consultation (Spidsberg, 2007; Spidsberg and Sorlie, 2012).

Despite the changes in social and political outlooks regarding sexual minorities and in the representation and participation of sexual minority members in the public sphere, labeling, intolerance, and discrimination toward the homosexual population is still common in society (Kama, 2014). It may be assumed that health professionals are not immune to these attitudes and messages. In Israel, the likelihood of lesbians receiving equitable perinatal nursing care ethically and medically has yet to be investigated. This issue is being considered in this study.

### Background

Social barriers have led to discrimination when accessing healthcare services, inadequate social programs for lesbian, gay, bisexual, transgender (LGBT) patients, and a dearth of knowledgeable caregivers (Sabin et al., 2015). Eliasson and colleagues noted that homophobia and heterosexualism are part of the socialization processes of health professionals (Eliason et al., 2011).

Hence, accepted institutional norms and values may obstruct this population’s pursuit of equality in the healthcare system and may also affect nurses’ perceived quality of care (Tzur Peled et al., 2019).

Negative attitudes toward lesbians are prevalent in the health care system (Dahl et al., 2013). In several studies, negative attitudes toward LGBT patients were found associated with the nurses’ unwillingness to provide care to this population. Nurses were apprehensive of saying or doing something wrong. These attitudes influenced their perceptions of quality of care to LGBT patients (Stonewall, 2015; Carabez and Scott, 2016; Dorsen and Van Devanter, 2016). Furthermore, a previous study indicated that the nurses’ perceptions of quality of care were affected by their attitudes toward perinatal care of LGBT patients. Nurses were prejudiced toward LGBTs during pregnancy, which biased their perceptions of the quality of care (Singer et al., 2019).

Behavioral intentions refer to the entire range of motivational factors reflecting how motivated the individual is to act (Ajzen, 1988). Behavioral intentions of caregivers toward sexual minorities have been examined in a qualitative study. Nurses noted that they were attentive to not harm LGBT patients (Beagan et al., 2012). Assessing nurses’ relationships and communication with lesbians may affect their satisfaction with the quality of care. For instance, in a study that evaluated the ability of 248 medical students to provide quality health care to LGBT patients, good communication between students and the LGBT patients was correlated with a higher level of treatment, expressed by obtaining a more generalized anamnesis (Sanchez et al., 2006).

### The Theoretical Framework

The present study was based on conceptualizations and components developed by Ajzen and Fishbein in their Theory of Reasoned Action (TRA) (Fishbein and Ajzen, 1975; Ajzen and Fishbein, 1980). This theory provides a framework for understanding and predicting the motivational effects on actual social behavior. TRA defines the associations between *beliefs, attitudes, norms, intentions, and behavior*, stating that a person’s behavior is determined by his or her behavioral intention of performing a certain behavior. The intention itself is determined by the person’s attitudes and subjective norms toward that behavior. More specifically, according to the TRA, interpersonal behavior is directly derived from behavioral intentions. Intention is the sum of all motivational factors that reflect the extent to which the individual displays action and how much effort that person is willing to invest (Ajzen, 1988). The intention itself evolves from the individual’s attitudes toward a certain behavior combined with his or her subjective norms (Ajzen, 1991).

Attitudes constitute one’s judgment regarding conducting a behavior and overall assessment of the same behavior. These attitudes are influenced by beliefs about the outcomes associated with the performance of a behavior and the value attributed to these outcomes (Ajzen, 1988; Gillmore et al., 2002).

The subjective norms of an individual represent his or her perception of the social environment’s attitudes toward that behavior (Colman, 2015). In other words the subjective norms are the result of his or her beliefs as to the extent to which “significant others” think that they must perform a certain behavior and the motivation to act according to their opinion (Colman, 2015; Glanz et al., 2015).

Studies where the TRA has been applied across contexts and disciplines such as health behavior (Rosemary et al., 2016), communication (Karnowski et al., 2017; Kim et al., 2020) and consumer behavior (Cobum and Farhar, 2004) provided support for our proposed research model, however, conceptualizations and variables derived from the TRA theory have yet not been used in studies dealing with nurses’ perceptions of the quality of perinatal health care offered to lesbians at women’s health centers. Therefore, our research question was: among are nurses from different ethnic groups perceptions of the quality of perinatal nursing care for lesbians related to their attitudes, subjective norms, behavioral intentions and assessment of relationships and communication?

### Study Purpose

It is important to note, that previous articles published in the context of the present study focused on the association between the nurses’ knowledge and attitudes toward lesbians receiving perinatal care (Tzur-Peled et al., 2019), the associations between the nurses’ professional and personal characteristics and their perceptions of their relationships and communication with lesbians seeking perinatal care (Tzur Peled et al., 2019).

Based on the TRA, our aims were to examine the associations between (a) nurses’ attitudes toward lesbians; (b) nurses’ attitudes relating to the care of lesbians; (c) nurses’ subjective norms; (d) nurses’ behavioral intentions to provide equal care; and (e) nurses’ assessment of relationships and communication as well as perceptions of the quality of perinatal care for lesbians amongst nurses from different ethnic groups.

## Materials and Methods

### Study Design

A cross-sectional study performed at women’s healthcare centers in Israel.

### Ethics Approval and Considerations

The study was approved by two Institutional Review Boards (Helsinki Committees) of Clalit Health Services (File number 0094-13-COM), Maccabi Health Services (File number 2015-10) and by the Ethics Committee of the Faculty of Health Sciences, Ben-Gurion University of the Negev, Israel (File number 2014-13).

The authors either emailed the questionnaires to nurses working in women’s healthcare centers or distributed them at staff meetings. An information sheet explaining the nature and importance of the study was attached to the questionnaire. Respondents were fully informed of the aims of the study and their right to refuse to participate or not answer any questions which made them feel uncomfortable.

### Population and Sample

All nurses (*n* = 270) who provided perinatal care to lesbians at different women’s healthcare centers in the community comprised the research population. Participants were recruited from all districts of the two largest health organizations in Israel. Of the 270 nurses approached, 184 completed a self-report questionnaire (a response rate of 74%). A similar response rate was reported in a previous study conducted amongst nurses in Israel (Kogan and Tabak, 2008). Possible reasons for not participating in the study were lack of time and unwillingness to collaborate with this type of study that might reveal rigid perceptions, homophobia, labeling and prejudice toward the lesbian community.

### Data Collection

Data were collected from nurses working at women’s health centers throughout Israel between December 2015 and April 2016. To avoid a potential selection bias, the researcher sent an email to the nursing staffs at the women’s health centers in all districts spread across the country of the Clalit Health Services and Maccabi Health Services.

One of the researchers (STP) attended staff meetings at the women’s health centers throughout the country, thus, ensuring that the sample obtained represented the intended population. Likewise, methodologically, it was necessary to overcome the possibility of biasing the findings due to social and professional desirability that make it difficult for health professionals to label, discriminate, and judge the LBGT population.

Therefore, we chose to examine the issue of behavioral intentions to provide equitable perinatal care to lesbians, the quality which would be comparable to the treatment that heterosexual women would receive, both directly and indirectly, by asking two questions: a direct question was “If you treat a lesbian woman in the future, do you think you will provide her with the same level of perinatal care that you provide to a heterosexual woman?” and an indirect question was “If another nurse on the staff were to treat a lesbian woman in the future, would you think she would provide her with the same level of perinatal care she provides to heterosexual women?” We believe this approach reduced the impact of the social and professional desirability of nurses to be perceived as providing uniform and equal care to all.

### Inclusion and Exclusion Criteria

The inclusion criteria included registered nurses who provide direct perinatal care to women at Health Maintenance Organizations (HMOs). Exclusion criteria were lack of knowledge of Hebrew and employed at a women’s healthcare centre for less than a year.

### Instrument Description

A pilot study was conducted amongst 42 well baby clinics nurses, to determine the reliability of the research instrument and the research process. The reliability of the questionnaires was found to be good (0.8–0.96).

### Nurses’ Attitudes Toward Lesbians and Gay Men’s Scale-ATLG (Herek, 1988)

The original questionnaire included 20 items: (a) 10 different attitudes toward gays (ATG); (b) 10 different attitudes toward lesbians (ATL). In our study, we used a sub-scale focusing on attitudes toward lesbians. Each item was answered using a four-point Likert scale from 1 (“strongly disagree”) to 4 (“strongly agree”). The overall score ranged from 10 to 40. A high score indicated negative attitudes. Based on this measurement tool, a dichotomous variable was constructed. A score of 10 indicated positive attitudes, scores > 10 indicated negative attitudes.

### The Gay Affirmative Practice Scale (Crisp, 2006)

Nurses’ attitudes toward the health care of homosexuals were measured by a 15-item questionnaire. Sample item: “In their practice with gay/lesbian clients, practitioners should support the different family structure of gay/lesbian families”. The overall score ranged from 15 to 60. A higher score indicated attitudes favoring positive action toward homosexuals. Based on this measurement tool, a dichotomous variable was created. A score ≥ 60 indicated positive attitudes and a score < 60 indicated negative attitudes.

### Subjective Norms

Our formulated questionnaire was based on the TRA (Fishbein and Ajzen, 1975; Ajzen and Fishbein, 1980), a tool for measuring the social impact on nurses in the context of providing care to lesbians. The questionnaire contained 12 statements. Each item was answered using a four-point Likert scale from 1 (“not at all”) to 4 (“strongly agree”). A high score indicated a great influence of significant others (e.g., family members, co-workers, and supervisors) in the nurse’s environment in the context of providing quality perinatal care to lesbians.

### Nurses’ Behavioral Intention to Provide Equal Perinatal Health Care to Lesbians

Based on Ben Natan and colleagues’ research (Ben Natan et al., 2009), direct behavioral intention was measured by the question: “Do you think you can commit to providing care to lesbians comparable to the standard care provided to heterosexual women?” To examine the nurses’ indirect behavioral intention, the same question was asked as to the potential care of lesbians by another staff nurse. Replies were provided on a five-grade Likert scale from 1 = “not at all” to 5 = “strongly agree”. The overall score ranged from 2 to 10. A high score indicated the behavioral intention of the nurse to provide lesbians the same quality care given to a heterosexual woman. A dichotomous variable was constructed regarding this variable, those who marked 5 = strongly agree, as opposed to those who marked 1–4 = disagree.

### A Nurse-Patient Relationship-Communication Assessment Tool (NPR-CAT) (Finch, 2006)

This 18-item questionnaire estimated the nurses’ perceptions of their relationships and communication with lesbian patients regarding six aspects: dominance, formality, exposure, availability, containment, and egalitarianism. The overall score ranged from 18 to 90. A high score indicated perceptions of good relationships and communication between the nurse and the patients.

### Socio-Demographic and Professional Background

This questionnaire comprised basic socio-demographic and professional background questions pertaining to age, ethnicity, level of religiosity, marital status, education, academic education, professional status, and residential area.

### Statistical Analysis

We hypothesized that positive associations would be found in the respondents’ attitudes toward lesbians regarding (a) their care, (b) subjective norms, (c) behavioral intentions, (d) assessment of relationships and communication, and (e) and perceptions of the quality of perinatal nursing care. The data were analyzed by the SPSS 21 program. Descriptive and inferential statistics and Pearson correlations examined the correlations among the study measures. A hierarchical multiple regression analyzed the data and identified the predictors of perceptions of the quality of perinatal nursing care for lesbians using the following independent variables: attitudes toward lesbians and their care, subjective norms, behavioral intentions, assessments of relationships and communication. *T*-tests were performed for sections “a,” “b,” “c,” and “d” to examine the differences in the average perceptions of the quality of perinatal care provided to the lesbians according to the independent variables. Pearson correlation examined the statistical correlation between section “e” and the nurses’ perceptions of the quality of perinatal care provided to the lesbians.

## Results

The participants’ mean age was 44.6 (SD = 9.66) and their mean nursing experience was 19 years (SD = 9.93). Demographics and participants’ characteristics are summarized in Table 1. The nursing population comprised Ashkenazi Jews of European origin (58%), secular (56%) and those residing in the centre of the country (51%). Most were married (86%) and were registered nurses with an academic degree in nursing (82.1%). Of those with a degree in nursing, 68% possessed a BA, 32% graduate degrees (MA and PhD) and 57% were graduates of post-basic nursing courses. Most (72%) worked in clinics and the remainder served as nursing directors at the clinics (Table 1).

**TABLE 1:** Personal and professional characteristics of the participants: categorical variables (*N* = 184).

Characteristic	Categories	Number	%
Ethnic origin	Ashkenazi Jews of European origin Sephardic Jews of Islamic origin Arab sector	107 59 18	58.2 32 9.8
Religiosity	Secular Traditional Religious including ultra-Orthodox	104 32 48	56.5 17.4 26.1
Marital status	Unmarried Married	25 159	13.6 86.4
Residential area	Central Israel Other areas (South, North, Jerusalem)	89 95	48.4 51.6
Education	Registered nurse Postgraduate registered nurse	33 151	17.9 82.1
Academic education	BA MA	107 50	68.2 31.8
Post basic course	Yes No	109 75	59.3 40.7
Professional status	Nurse director Clinic nurse	51 133	27.8 72.2

Approximately 75% were acquainted with a lesbian. Of all the participants, only 17.9% indicated that they had undergone vocational training as to the health, psychological, and social characteristics of lesbians. Of these, 66.7% thought that they had been provided with professional tools for working and treating lesbians. Of the nurses who participated in the study, 60.4% disagreed or were uncertain that exposure to the sexual orientation of the patients was important when treating the women (Table 2).

**TABLE 2:** Nurses’ acquaintance with lesbians: categorical variables (*N* = 184).

Characteristic	Categories	Number	%
Personal acquaintance with lesbians	Low Moderate High Unknown	44 62 73 5	23.9 33.7 39.7 2.7
Professional training	Yes No Uncertain Unknown	10 151 21 2	5.4 82.1 11.4 1.1
Continuing education program	Yes No Uncertain Unknown	33 142 7 2	17.9 77.2 3.8 1.1
Professional tools	Yes No Uncertain	22 6 5	66.7 18.2 15.1
Importance of exposure to patient’s orientation	Agree Disagree Uncertain Unknown	71 59 52 2	38.6 32.1 28.3 1.0

Most of the nurses (76.1%) held negative attitudes toward lesbians and negative attitudes toward the care of lesbians (83.2%). Approximately half the nurses had a low subjective norm advocating for the provision of unequal perinatal care to lesbians and 64% responded that they fully agree with the principle of equal care for lesbian patients (Table 3).

**TABLE 3:** Attitudes, subjective norms, and nurses’ behavior intention-frequencies and percentages (*N* = 184).

Characteristic	Categories	Number	%
Nurses’ attitudes toward lesbians	Positive attitudes Negative attitudes	44 140	23.9 76.1
Nurses’ attitudes toward lesbians’ care	Positive attitudes Negative attitudes	31 153	16.8 83.2
Subjective norms regarding the perinatal nursing care of lesbians	High norms Low norms	88 95	47.8 52.2
Behavioral intention	No	61	35.9
	Strongly agree	118	64.1

To examine the research model and as a basis for hierarchical regression analysis, an initial examination of relationships between the various research variables was performed using the Pearson correlation (Table 4).

**TABLE 4:** Pearson correlation coefficient between study variables (*N* = 184).

		1	2	3	4	5	6
1	Nurses’ attitudes toward lesbians’ care						
2	Nurses’ attitudes toward lesbians	0.40***					
3	Subjective norms	0.34***	0.33***				
4	Behavioral intention	0.11	0.18*	0.08			
5	Relationships and communication	0.51***	0.57***	0.31***	0.02		
6	Perceptions of quality of care	0.50***	0.45***	0.37***	3.0	0.73***	

*p < 0.05, ***p < 0.001.

Table 4 shows a positive correlation between nurses’ attitudes toward lesbians’ care and their attitudes toward lesbians. Thus, the more positive the nurses’ attitudes toward lesbians’ care, the more positive their attitudes toward lesbians. Likewise, significant correlations were found between nurses’ attitudes toward lesbians’ care and the subjective norm, relationships, communication, and perceptions of the quality of care. Namely, the more positive the nurses’ attitudes toward lesbians’ care, the higher the subjective norm, the relationship, communication, and the perception of quality of care. Furthermore, regarding the nurses’ attitudes toward lesbians, significant positive correlations were found between subjective norm, behavioral intention, relationship, communication, and perception of quality of care. The correlation between subjective norm and behavioral intention is significant, yet, lower compared to other correlations. Therefore, the higher the nurses’ attitudes toward lesbians, the higher the subjective norms, behavioral intention, relationships, communication, and perception of quality of care. Finally, a high positive correlation was found between relationships, communication and perception of quality of care. Specifically, the better the quality of the relationship and communication, the higher the quality of care perceived by the nurses. Herein, using a multiple hierarchical regression model, we examined the contribution of the variables found significant in the univariate analysis explaining the differences in perceptions of quality of care. The following variables were included in the multiple hierarchical regression model: ethnic origin, religiosity, acquaintance, attitudes toward lesbians’ care, attitudes toward lesbians, subjective norms, and relationships and communication (Table 5).

**TABLE 5:** Hierarchical multiple regression analysis explaining the variability of perception of quality of perinatal nursing care the nurses provided the lesbians (*N* = 184).

Step	Variable	*B*	*SE B*	β	Δ*R*^2^	*R* ^2^
1					0.08**	0.08**
	Ashkenazi Jews of European origin	4.53	2.35	0.25*		
	Sephardic Jews of Islamic origin	1.15	2.44	0.06		
	Religiosity	–1.33	0.69	−0.15*		
2					0.04**	0.12***
	Ashkenazi Jews of European origin	2.01	2.50	0.11		
	Sephardic Jews of Islamic origin	–1.66	2.60	–0.09		
	Religiosity	–0.75	0.70	–0.08		
	Acquaintance	2.63	0.92	0.23*		
3					21***.	0.33***
	Ashkenazi Jews of European origin	–1.87	2.30	–0.10		
	Sephardic Jews of Islamic origin	–4.83	2.40	−0.25*		
	Religiosity	0.97	0.70	0.11		
	Acquaintance	1.23	0.83	0.11		
	Attitudes toward lesbians’ care	5.0	0.99	0.35**		
	Attitudes toward lesbians	0.49	0.12	0.32**		
4					0.00	0.33***
	Ashkenazi Jews of European origin	–1.91	2.32	–0.11		
	Sephardic Jews of Islamic origin	–4.80	2.40	−0.25*		
	Religiosity	0.10	0.71	0.11		
	Acquaintance	1.21	0.83	0.11		
	Attitudes toward lesbians’ care	5.03	1.07	0.35		
	Attitudes toward lesbians	0.47	0.13	0.31**		
	Subjective norm	0.36	0.98	0.03		
5					0.23***	0.56***
	Ashkenazi Jews of European origin	–1.60	1.90	–0.09		
	Sephardic Jews of Islamic origin	–3.44	1.10	–0.18		
	Religiosity	1.18	0.57	0.13*		
	Acquaintance	0.78	0.68	0.07		
	Attitudes toward lesbians’ care	2.10	0.92	0.15*		
	Attitudes toward lesbians	0.07	0.12	0.050		
	Subjective norm relationships and	0.93	0.80	0.070		
	communication	9.92	1.07	0.63**		

*p < 0.05, **p < 0.01, ***p < 0.001.

Table 5 presents the hierarchical regression model explaining the variability in nurses’ perceptions of quality of care provided to lesbians the table demonstrates that the various indices together contributed 56% to the explanation of the variance in the perception of quality of care. Initially, the indices of origin and religiosity were introduced, explaining 8% of the variance. A significant beta was found for participants of Ashkenazi origin, consequently, amongst these nurses the perception of the quality of care was better compared with the nurses of Sephardic and Islamic origin. In addition, a negative and significant beta coefficient was found for religiosity, therefore, amongst the secular nurses, the perception of quality of care was higher. Secondly, the nurses were familiarized with the lesbians’ index which contributed an additional 4% explanation of the variance. The beta coefficient of this index was positive, therefore, the higher the acquaintance, the better the nurses’ perception of the quality of care. Thirdly, two attitude indices were introduced, i.e., attitudes toward lesbians’ care and attitudes toward lesbians, contributing 21% to explain the variance. The beta coefficients of these attitudes were positive, therefore, the more positive the nurses’ attitudes, the better their perception of quality of care. Fourthly, the subjective norm index was introduced, yet did not contribute to the explanation of the variance. In the fifth and final step, the variable perception of relationships and communication was introduced. This variable contributed another 23% to explain the variance. The beta coefficient was positive, so the better the relationship and communication, the better the nurses’ perception of the quality of care.

It appears that although in the third and fourth steps, a significant contribution to the independent variable was found for the two attitudes, the beta coefficients of these attitude variables were reduced and became insignificant at the fifth step, when the variable of relationship and communication was introduced. This finding suggests the possibility of mediation, i.e., that the quality of the relationship and communication mediated between attitudes toward lesbians’ care and toward lesbians; and nurses’ perception of the quality of care provided to the lesbians. Consequently, the more positive the nurses’ attitudes, the more positive their perception of the relationships and communication leading to an improvement in the perception of quality of care. As mentioned, the fifth step explained 56% of the variability in the perception of relationships and communication.

The first hypothesis: nurses’ attitudes toward lesbians and their perceptions of quality of care were examined by a *t*-test. The perceptions of quality of care were significantly higher amongst nurses with positive attitudes toward lesbians than those with negative attitudes (*t* = 3.07; *df* = 182; *p* = 0.000).

The second hypothesis: nurses’ attitudes toward perinatal care of lesbians, and their perceptions of quality of care were tested by a *t*-test. The perceptions of quality of care were significantly higher amongst nurses with positive attitudes toward perinatal care of lesbians than those with negative attitudes (*t* = 4.51; *df* = 182; *p* = 0.000).

The third hypothesis: nurses’ subjective norm concerning perinatal care for lesbians, and their perceptions of quality of care were tested by a *t*-test. The perceptions of quality of care were significantly higher amongst nurses with a high subjective norm concerning perinatal care for lesbians compared to those with a low subjective norm in relation to perinatal health care for lesbians (*t* = 5.23; *df* = 181; *p* = 0.000).

The fourth hypothesis: nurses’ behavioral intention to provide equal perinatal care to lesbians and their perceptions of quality of care were tested by a *t*-test. The mean perception of the quality of perinatal care provided to lesbians was significantly higher amongst nurses with a high behavioral intention to provide equal perinatal care to lesbians than those with low behavioral intentions (*t* = 3.50; *df* = 177; *p* = 0.004).

The fifth hypothesis: nurses’ assessment of their relationships and communication with lesbians and their perceptions of quality of care were tested using the Pearson correlation. A high correlation (0.73, *p* < 0.001) was found between the nurses’ assessment of their relationship and communication with lesbians and their perception of quality of care. Specifically, the more likely that the relationship and communication with the lesbian patients are evaluated, the better the perception of quality of care.

## Discussion

Our research question was reinforced by our findings. The analysis revealed that attitudes of nursing staff members from different ethnic groups, subjective norms, behavioral intentions, assessments of relationships and communication were linked with their perceptions of the quality of perinatal care provided to lesbians. To assess the research model and as a basis for the hierarchical regression analysis, an initial examination of correlations between the various research variables was performed, using the Pearson correlation coefficient. The hierarchical regression analysis demons that several indices together contributed 56% to the variability in the nurses’ perception of quality of care. The relationship and communication assessment variable contributed 23% for predicting the perception of quality of care. Other variables that contributed for predicting the perception of quality of care were nurses’ ethnic origin and degree of religiosity (8%), acquaintance (4%) and nurses’ attitudes toward lesbians’ care and toward lesbians (21%). A possible explanation for the significant contribution of the variable relationship and communication for predicting the perception of quality of care is that quality care provided to lesbians is based on relationships and communication, characterized by openness, sensitivity, involvement, and support (Erlandsson et al., 2010).

The use of conceptualizations and variables of the TRA in understanding nursing staff members’ attitudes, subjective norms, behavioral intentions, assessment of their relationships, communication and perceptions of the quality of perinatal care provided to lesbians is innovative and thus far, no studies have examined these correlations. Furthermore, the variables of the TRA were found to predict nurses’ perceptions of the quality of care. Likewise, the results of the present study add an important layer to the strong correlation between the nurses’ assessment of their relationships and communication with lesbians and their perceptions of the quality of perinatal nursing care. It appears that the assessment of the relationships and communication with lesbians plays a significant role in the nurses’ perceptions of the quality of perinatal care provided to lesbians.

### Nurses’ Attitudes Toward Lesbians and Their Perceptions of Quality of Care

Nurses with positive attitudes toward lesbians reported a significantly higher quality of care than nurses with negative attitudes. This finding is consistent with several previous studies which demonstrated that the nurses’ attitudes to lesbians influenced their perceptions of the quality of caregiving (Albuquerque et al., 2016). The literature notes that nurses have often demonstrated disdain for LGBT patients (Dorsen and Van Devanter, 2016). Tzur Peled et al. (2019) found that some of the nurses felt conflicted and dissonant with their attitudes toward lesbians, and the need and desire to be tolerant in the context of perinatal care for these patients. In other studies, the negative attitudes of nurses toward LGBTs were correlated with their unwillingness to provide health care to members of this community (Marques et al., 2015). These attitudes influenced the nurses’ perceptions of the quality of care they provided (Carabez and Scott, 2016).

### Nurses’ Attitudes Toward Perinatal Health Care of Lesbians, and Their Perceptions of Quality of Care

A previous study has shown that the nurses’ perceptions of quality of care were affected by their attitudes toward perinatal care of LGBTs. The participants expressed misconceptions, prejudice, insensitive language, and lack of normativity regarding LGBT pregnant patients, which biased their perceptions of the quality of care they had provided (Singer et al., 2019). On the other hand, a qualitative study examining the attitudes of specialist nurses from the United States regarding the care given to gays and lesbians, found that some of the nurses displayed positive attitudes and others reported ambivalence toward the health needs of this population. In their assessment, their attitudes cast a shadow on their perceptions of quality of care (Dorsen and Van Devanter, 2016). However, in a qualitative study, the nurses found that their patients’ sexuality was irrelevant to them and that they believed that the care provided to all their patients was qualitative and egalitarian (Beagan et al., 2012). These studies indicate a lack of uniformity which may be due to differences in factors such as the type of population, work settings and countries.

### Nurses’ Subjective Norms as to Perinatal Care for Lesbians and Their Perceptions of Quality of Care

The perceptions of quality of care were significantly higher amongst nurses with a high subjective norm as to perinatal care for lesbians compared to nurses with a low subjective norm. This finding underscores the meaningful impact of significant others (e.g., family members, co-workers, and supervisors) in the nurse’s environment on the averages of perceptions of the quality of perinatal care for lesbians. This finding is consistent with previous studies. The presence of heterosexual norms in the caregiver system contributed to discomfort and even influenced the perceptions of quality of care amongst the nurses (Dahl et al., 2013).

### Nurses’ Behavioral Intention to Provide Equal Perinatal Health Care to Lesbians and Their Perceptions of Quality of Care

The mean score for perceptions of the quality of perinatal health care for lesbians was significantly higher amongst nurses with a high behavioral intention to provide equal perinatal health care to lesbians than amongst nurses with a low behavioral intention. This finding is consistent with a previous study in which the intention of the hospital nursing staffs to provide quality care to drug addicts, predicted high quality perceptions (Albuquerque et al., 2016). A possible explanation of the current study’s finding is that disproportionate attitudes amongst the treating staff may lead to unequal care expressed by hostility and interpersonal distance from lesbians. It is possible that some of the nurses were conflicted between their personal tendency to take a judicial approach, and the demands of the nursing role, derived from the moral value system that characterizes the nursing profession. This conflict may have impaired the nurses’ perception of quality of care (Tillman et al., 2016).

### Nurses’ Assessment of Their Relationship and Communication With Lesbians and Their Perceptions of Quality of Care

The nurses’ assessment of their relationships and communication with lesbians was correlated with their perceptions of the quality of perinatal nursing care. Namely, the higher the scores when evaluating the relationships and communication with lesbian patients, the higher the prevalence of the nurses’ perceptions of the quality of perinatal care for lesbians. This finding was supported by Radix and Maingi who found that when nurses felt uncertain as to the proper way to address lesbian patients, their discomfort affected relationships and communication as well as their perceptions of the quality of care (Radix and Maingi, 2018).

## Conclusion

This study is innovative as it investigated a sensitive topic in Israeli society. The results advance important policy implications. The study revealed that there are nurses providing perinatal care in community clinics in Israel who retain negative attitudes toward lesbians and their care. There are increasing numbers of parents of the same sex who deserve to be cared for by health professionals who convey an attitude of equality. We showed that there is still a great distance to go to achieve this equality. The importance of this study is that it highlights the fact that even professionals must invest in improving their awareness of their own prejudices and become more aware of the limitations of their power and responsibility in accepting minorities. The authors, therefore, stress the importance of formulating a formal policy in the field of LGBT medicine in Israel.

### Theoretical and Practical Implications

There are several important implications of this study. On a theoretical level, this pioneer study, for the first time, assesses the perceptions of nurses working in women’s health centers as to the quality of the perinatal care provided to lesbians, thus, contributing to the body of knowledge in terms of lesbians’ visibility in the health system.

The use of concepts and variables from the TRA to examine the correlations between personal characteristics, attitudes, subjective norms, behavioral intentions, and nurses’ perception of the quality of care provided to lesbians, is innovative and adds an important layer to the original model of the theory (Fishbein and Ajzen, 1975; Ajzen and Fishbein, 1980). Additional research is vital in order to better understand the status of lesbians in the healthcare system. Furthermore, it is extremely important to change stereotypic perceptions of the lesbian population and to improve their communication skills when providing healthcare. This can be achieved by providing educational programs dealing with the unique biopsychosocial needs of lesbians to all staff nurses and nursing students. Similarly, we hope that this study will assist in raising the awareness of healthcare authorities as to the special health needs of this population.

### Limitations

This study has several limitations. Firstly, the study does not provide a strong justification for the TRA for the theoretical underpinning of the model, although in the existing literature TRA/TPB have been applied across contexts and disciplines and have provided support for our proposed model (Ramkissoon et al., 2012, 2013; Megeirhi et al., 2020; Ramkissoon, 2020, 2021).

The second limitation lies in the research tool used to measure the behavioral intention to provide equal perinatal care to lesbians. The mean score of our behavioral questionnaire was very high; 4.60 out of a 1–5 score range. Specifically, the behavioral intention variance was limited. It is possible that the nurses who participated in the study were reluctant to respond honestly and acknowledge behavior that is not professionally acceptable, e.g., providing inequitable care for all. The third limitation relates to the type of study which was cross-sectional. It is possible that longitudinal research at several points of time would have yielded data that would have broadened the understanding of the nurses’ perceptions of the quality of care. The fourth limitation is the fact that the research population did not include the lesbians themselves, relying only on the nursing staff reports without exploring the experiences of the lesbian patients.

The small size of the sample and furthermore, the fact that it included only two health organizations are also limitations of this study. We were also unaware as to whether lesbians were amongst the nurses themselves. Finally, only subjective variables were included with no hard data from an objective source.

## Data Availability Statement

The original contributions presented in the study are included in the article/supplementary material, further inquiries can be directed to the corresponding author.

## Ethics Statement

The studies involving human participants were reviewed and approved by the study was approved by two Institutional Review Boards (Helsinki Committees) of Clalit Health Services (File number 0094-13-COM), Maccabi Health Services (File number 2015-10) and by the Ethics Committee of the Faculty of Health Sciences, Ben-Gurion University of the Negev, Israel (File number 2014-13). The patients/participants provided their written informed consent to participate in this study.

## Author Contributions

ST-P, TK, and OS: study design, data analysis, manuscript writing, and critical revisions for important intellectual content. ST-P: data collection. TK and OS: study supervision. All authors contributed to the article and approved the submitted version.

## Conflict of Interest

The authors declare that the research was conducted in the absence of any commercial or financial relationships that could be construed as a potential conflict of interest.

## Publisher’s Note

All claims expressed in this article are solely those of the authors and do not necessarily represent those of their affiliated organizations, or those of the publisher, the editors and the reviewers. Any product that may be evaluated in this article, or claim that may be made by its manufacturer, is not guaranteed or endorsed by the publisher.
